# Tartarized Antimony in Traumatic Pneumonitis and Pleuritis

**Published:** 1864-09

**Authors:** P. W. Douglas

**Affiliations:** Surgeon 2d Regiment Georgia State Line.


**Art. VII.**—*Tartarized Antimony in Traumatic Pneumonitis and Pleuritis.* By P. W. Douglas, Surgeon 2d Regiment Georgia State Line.

Thomas Tolant, private company "A," 2d regiment Georgia State Line, was stabbed with a pocket knife three inches in length, in the chest, between the third and fourth ribs, penetrating the pleura and slightly puncturing the lungs. Loss of blood externally very slight; considerable hemorrhage internally; nervous depression very great, but of short duration; reaction violent; within twelve hours, or by the next morning after the occurrence, at eight o'clock the previous evening, found a high state of inflammation, and hurried and oppressed respiration, almost incapable of articulation, entirely monosyllabic. The chest, on the side which received the wound, was considerably swollen, giving a drum-like sound on percussion and the quill sound on auscultation; the opposite or right side, the rustling and crepitant sound. Pulse, 140 to 150; skin hot and dry; tongue dry, though not thirsty. This case, altogether, presented a very unfavorable prognosis. I at once put him upon an anodyne diaphoretic of tartarized antimony, swt. spts. nitre and morphine. In about six or eight hours the whole surface of the body was in a gentle perspiration, and pulse, though equally full, was yet more compressible. After using the antimonial pulv. for six or eight hours, I desisted, and put him upon veratrum, nitre and morphine, with every caution to his intelligent nurse to watch his pulse and inform me. About midnight I was awakened and told that my patient was thought to be growing very weak; pulse, very small and slow. I simply ordered a little toddy and discontinuance of the veratrum. Next morning, six hours after stopping veratrum, found pulse gradually rising and patient spitting up some blood. Ordered saline purge. After operating, about ten o'clock of the second day, again put him upon the tartarized emetic mixture for two days and nights unintermittingly, stomach and bowels tolerating it well. He took nothing else, and every day I could plainly perceive a subsidence of the active inflammation in the softening pulse, cool and moist skin, and gentle and unoppressed respirations. He is now well. It is evident to my mind, if I had abstracted that amount of blood which would have been necessary to reduce the pulse and subdue the acute inflammatory symptoms, my patient would have died, because of reasons known to every medical man who has a local injury, a considerable amount of extravasated blood and great nervous depression to treat. Tartar emetic is no new remedy in conditions like the one I have written, but for its uniform, satisfactory and immediate success, I give it to you, that forsooth it may be another atom of rational medicine placed in the long since overbalanced scale against the lancet in inflammatory affections of the chest.

